# Weekly dynamics, subtype replacement and regional heterogeneity of influenza activity in China, 2024–2026: A national sentinel surveillance analysis

**DOI:** 10.1371/journal.pone.0361588

**Published:** 2026-11-04

**Authors:** Simin Wang, Zhi Xia, Buyun Shi, Ming Chen, Xiaolan Shu, Pan Peng

**Affiliations:** 1 Department of Maternal and Child Health Hospital of Hubei Province, Hubei University of Medicine, Shiyan City, Hubei Province, China; 2 Department of Pediatric Intensive Care Unit, Maternal and Child Health Hospital of Hubei Province, Wuhan, Hubei, China; 3 Children’s Infectious Disease Prevention and Control Center of Hubei Province, Wuhan, Hubei, China; 4 Department of Dermatology, Maternal and Child Health Hospital of Hubei Province, Wuhan, Hubei, China; University of Brunei Darussalam: Universiti Brunei Darussalam, BRUNEI DARUSSALAM

## Abstract

**Objective:**

National influenza surveillance in China is reported weekly, but recent post-pandemic patterns integrating regional timing, subtype/lineage replacement and outbreak activity have not been quantitatively characterized in a continuous national series. We aimed to reconstruct and quantify weekly influenza dynamics in China from 2024/W01 to 2026/W21.

**Methods:**

We performed a retrospective secondary time-series analysis of 125 publicly available, aggregate weekly reports from the Chinese Center for Disease Control and Prevention and Chinese National Influenza Center. Values were reconciled against official reports when working datasets differed or quality-control checks identified inconsistencies. Positivity was recalculated from positive and tested specimens. Regional differences were evaluated across paired weeks using heteroskedasticity- and autocorrelation-consistent Newey-West standard errors and exploratory lead-lag relationships were assessed using cross-correlation of first-differenced weekly series.

**Results:**

Nationally, 1,796,636 influenza-like illness specimens were tested and 253,219 were influenza-positive (weighted positivity, 14.1%); national positivity peaked in 2025/W49 at 49.8%. Overall weighted positivity was 14.6% in Southern China and 13.3% in Northern China. Across 125 paired weeks, Southern China had a mean positivity 4.41 percentage points higher than Northern China (95% CI, 2.23–6.59; P < 0.001) and a mean ILI percentage 0.77 points higher (95% CI, 0.48–1.05; P < 0.001). First-difference positivity changes were most strongly correlated when Northern China led Southern China by one week (r = 0.781). Among 253,104 explicitly reported subtype/lineage detections, A(H3N2) was largest (45.6%), followed by A(H1N1)pdm09 (33.8%) and B(Victoria) (20.6%). Predominance followed A(H3N2) in 2024/W01, B(Victoria), A(H1N1)pdm09, A(H3N2) and B(Victoria) again in early 2026. Reported outbreaks peaked in 2025/W48 at 1,541, during A(H3N2) predominance.

**Conclusions:**

Recent influenza activity in China was multi-wave and regionally heterogeneous, with measurable north-south temporal differences, sequential subtype replacement and a large A(H3N2)-associated outbreak surge. The analysis also demonstrates the importance of explicit source-level quality control when reusing aggregated public surveillance reports.

## Introduction

Seasonal influenza remains a major cause of respiratory illness and preventable morbidity worldwide. The World Health Organization estimates that seasonal influenza causes around one billion infections annually, including 3–5 million severe cases and 290,000–650,000 respiratory deaths [1,2]. Children and other high-contact populations contribute substantially to transmission and influenza remains an important cause of acute lower respiratory infection and hospitalization in young children [3]. Beyond individual morbidity, influenza waves affect outpatient visits, emergency care, hospital capacity, school functioning and vaccination programs [4]. Continuous virological surveillance through systems such as the Global Influenza Surveillance and Response System provides essential evidence for vaccine strain selection and epidemic preparedness [5].

The COVID-19 pandemic disrupted influenza circulation in many settings. Non-pharmaceutical interventions, including school closure, masking, physical distancing, travel restrictions and reduced social mixing, were associated with historically low influenza activity [6]. In China and the United States, these interventions reduced influenza transmission and altered subtype patterns [7]. Other evidence from China also showed that influenza activity and circulating subtypes changed substantially during the COVID-19 period [8]. After relaxation of these interventions, influenza viruses re-entered populations with changed exposure histories and accumulated susceptibility, often described as an immunity gap or immunity debt [9]. The two-peak influenza epidemics reported in China in 2023 further illustrated that post-pandemic circulation could differ from a simple pre-pandemic winter-dominant pattern [10].

Previous studies have described influenza seasonality, spatial heterogeneity and subtype circulation in China. Pre-pandemic analyses showed distinct seasonal patterns across northern, southern and subtropical regions, with implications for vaccination timing [11]. Other studies characterized transmission patterns of seasonal influenza subtypes in China [12] and longer-term spatiotemporal heterogeneity across mainland China [13]. Recent global and multi-country surveillance analyses have also documented altered influenza activity and subtype circulation after the COVID-19 pandemic [14,15]. However, recent Chinese weekly surveillance signals – virological positivity, regional timing, subtype/lineage replacement and outbreak activity – have rarely been quantified together in a single continuous post-2023 series. Publicly released aggregate reports also require careful distinction between influenza-positive surveillance specimens and national case counts and their reuse raises reproducibility questions when source tables contain internal inconsistencies.

To address these gaps, we reconstructed a national weekly dataset from official China CDC and CNIC surveillance reports [16]. We aimed to quantify weekly influenza activity from 2024/W01 to 2026/W21, compare Southern and Northern China using paired-week inference and exploratory lag analysis, characterize subtype/lineage replacement, summarize outbreak activity and explicitly document source-level data discrepancies. Calendar-year summaries were retained for descriptive comparability; sensitivity summaries were also constructed using the W40-W39 influenza-season convention.

## Materials and methods

### Study design, data source and scope

We conducted a retrospective secondary time-series analysis of publicly available, aggregate national influenza sentinel-surveillance reports. All epidemiological and virological data were obtained from weekly reports released by the Chinese Center for Disease Control and Prevention (China CDC) and the Chinese National Influenza Center (CNIC) [16]. These reports summarize influenza-like illness (ILI) surveillance and virological testing from the national sentinel surveillance network. The investigators did not recruit participants, access individual-level clinical records or identifiers, or handle the biological specimens underlying the surveillance reports.

The study period covered 2024/W01 to 2026/W21, corresponding to 125 consecutive epidemiological weeks. Epidemiological weeks are denoted in year/week format (YYYY/Www) throughout the manuscript. Regional analyses followed the surveillance-report classification of Southern and Northern China. Province-level data were not available in the reconstructed series. Calendar-year summaries refer to calendar years and were not interpreted as formal influenza seasons. To address season-definition concerns, a sensitivity summary used the W40-W39 convention; because the observation window began in 2024/W01 and ended in 2026/W21, only 2024/W40-2025/W39 was a complete W40-W39 interval.

### Variables and definitions

Weekly variables included ILI percentage in Southern and Northern China; tested ILI specimens; influenza-positive specimens; influenza A and B detections; A(H1N1)pdm09, A(H3N2), A untyped, B(Victoria), B(Yamagata) and B untyped detections; and reported influenza outbreaks by etiological classification. Because the public reports contain aggregate laboratory surveillance outputs, we use the term influenza-positive specimens rather than influenza cases.

For all analytical comparisons, weekly positivity was recalculated as influenza-positive specimens divided by tested specimens multiplied by 100, using the unrounded value for peak identification and statistical analyses. Reported percentages were retained only for source verification. Weekly predominant subtype/lineage was defined as the explicitly reported subtype/lineage detection with the largest count in the corresponding national or regional stratum. Consecutive weeks with the same national predominant subtype/lineage were grouped into predominant periods.

### Data extraction, reconciliation and quality control

Two structured working files derived from the official weekly reports were compared field-by-field. The final dataset followed a prespecified source hierarchy: when the working files differed, when automated arithmetic checks identified inconsistencies, or when a reconstructed subtype value represented a residual rather than an explicitly reported detection, the corresponding China CDC/CNIC weekly report was rechecked and the explicitly reported value was used. Residual subtype counts were not imputed in the revised dataset.

Quality-control checks evaluated week continuity, positive specimens versus influenza A plus B totals, type totals versus subtype/lineage detections, national totals versus the sum of Southern and Northern values, outbreak totals versus etiological outbreak categories and reported versus recalculated positivity. When an official report was internally inconsistent, we retained the explicitly reported values at the relevant analysis level rather than forcing arithmetic reconciliation; national analyses used national values and regional analyses used the corresponding regional values. These discrepancies and their handling are documented in S2 Table. No weekly observation was excluded.

### Ethics statement

This secondary analysis used only publicly accessible aggregate surveillance summaries. The authors did not access participant-level data, clinical records, identifiers or human biological specimens. Although the underlying national surveillance system includes specimen collection as part of public health surveillance, this study analyzed only published aggregate outputs. Institutional review board approval and individual informed consent were therefore not applicable to this secondary analysis.

### Statistical analysis

Weekly trends were summarized using line plots, stacked area plots, heatmaps and stacked bars. Calendar-year and overall summaries were calculated for 2024, 2025, 2026/W01-W21 and the full study period. Weighted positivity was calculated from summed positive and tested specimens. Mean weekly ILI percentages were calculated separately by region. Subtype/lineage composition used the sum of explicitly reported subtype/lineage detections as the denominator; this denominator can differ slightly from total influenza-positive specimens when official reports do not provide arithmetically complete subtype classification.

To quantify regional heterogeneity without treating individual specimens as independent observations, Southern-minus-Northern differences in weekly positivity and weekly ILI percentage were analyzed across 125 paired epidemiological weeks. Mean paired differences were estimated using intercept-only ordinary least-squares models, for which the intercept represents the mean weekly South-minus-North difference. Because adjacent weekly surveillance observations may exhibit heteroskedasticity and serial correlation, inference was based on heteroskedasticity- and autocorrelation-consistent Newey-West standard errors [17]. A maximum lag of 4 weeks was selected to account for approximately one month of short-term serial dependence; sensitivity analyses using maximum lags of 8 and 12 weeks were also performed.

Exploratory regional lead-lag relationships were assessed using cross-correlation of first-differenced weekly series to reduce correlation attributable to shared low-frequency epidemic trends [18]. The primary lag-search window was restricted to −8 to +8 weeks to focus on short-term regional temporal offsets within approximately two months rather than longer-term seasonal relationships. Sensitivity analyses used alternative + /-4-week and +/-12-week search windows. Positive lags indicate that changes in Northern China preceded changes in Southern China. These analyses were exploratory and were not interpreted causally. Formal forecasting, province-level spatial modeling and climate or mobility modeling were not performed because the dataset contained only two broad regional strata and approximately 2.4 years of observation. Analyses were performed in Python 3.13.5 using NumPy 2.3.5, statsmodels 0.14.6 and Matplotlib 3.10.8.

### Use of artificial intelligence tools

DeepSeek and ChatGPT (OpenAI) were used for language editing and organizational assistance during manuscript preparation and revision. All AI-assisted outputs were reviewed and verified by the authors against the source data; the authors made all scientific interpretations and conclusions and take full responsibility for the manuscript.

## Results

### Dataset overview and national influenza activity

The final dataset included 125 consecutive weekly surveillance records from 2024/W01 to 2026/W21 (S1 Dataset). During the study period, 1,796,636 ILI specimens were tested nationally and 253,219 were influenza-positive, yielding a weighted national positivity of 14.1% (Table 1). National positivity followed a multi-wave pattern and peaked in 2025/W49 at 49.8%, when 11,138 of 22,381 tested specimens were positive. In 2024, 561,595 specimens were tested and 70,339 were positive (12.5%); in 2025, 868,497 were tested and 133,291 were positive (15.3%); and in 2026/W01-W21, 366,544 were tested and 49,589 were positive (13.5%).

**Table 1:** National summary of influenza surveillance indicators.

Period	Weeks	Tested specimens	Positive specimens	Weighted positivity (%)	Peak positivity week	Peak positivity (%)	Outbreaks
2024	52	561,595	70,339	12.5	2024/W01	44.4	903
2025	52	868,497	133,291	15.3	2025/W49	49.8	7,519
2026/W01-W21	21	366,544	49,589	13.5	2026/W01	26.0	379
Overall	125	1,796,636	253,219	14.1	2025/W49	49.8	8,801

**Note.** Values for 2026 cover weeks 1–21 only. Weighted positivity was calculated as positive specimens divided by tested specimens.

The highest ILI percentage occurred in 2025/W49 in Southern China (11.1%) and 2025/W48 in Northern China (9.1%), rather than in early 2026. Thus, the principal 2025 virological and syndromic peaks were temporally close but are reported separately because ILI percentage and laboratory positivity measure different surveillance constructs (Fig 1).

**Fig 1:**
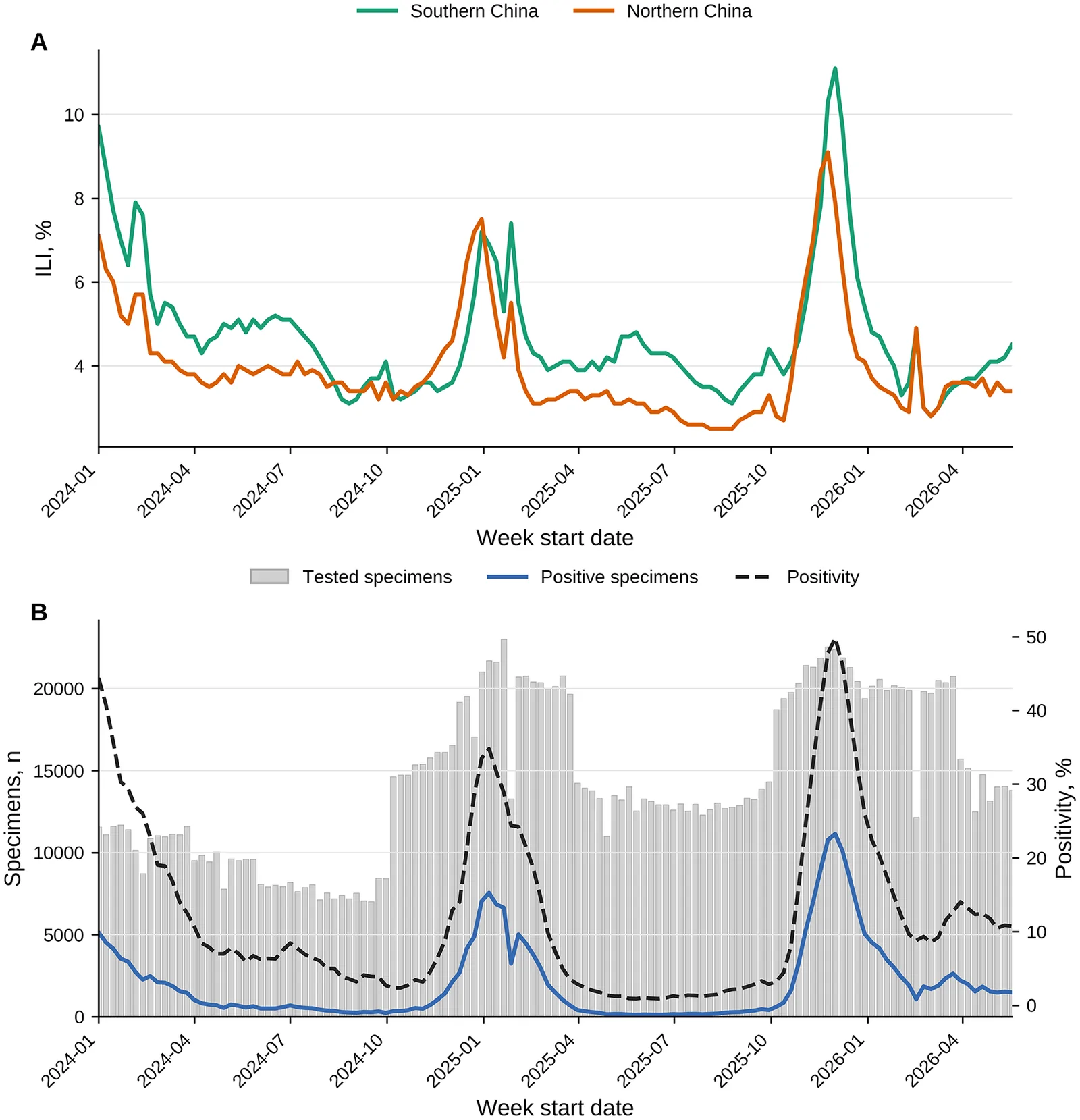
Weekly influenza activity in China. Panel A shows weekly influenza-like illness (ILI) percentages in Southern and Northern China from 2024/W01 to 2026/W21. Panel B combines national tested specimens (gray bars), influenza-positive specimens (blue solid line) and recalculated positivity (black dashed line).

Using the W40-W39 convention as a sensitivity summary, the complete 2024/W40-2025/W39 interval included 806,385 tested specimens, 77,719 positives (weighted positivity, 9.6%), A(H1N1)pdm09 predominance and 1,033 reported outbreaks. The later 2025/W40-2026/W21 interval was incomplete but had higher weighted positivity (19.7%), A(H3N2) predominance and 7,438 reported outbreaks (S3 Table).

### Regional heterogeneity between Southern and Northern China

Across the full study period, Southern China contributed 1,078,477 tested specimens and 157,732 positive specimens (weighted positivity, 14.6%), whereas Northern China contributed 718,168 tested specimens and 95,487 positive specimens (13.3%) (Table 2). Mean weekly ILI percentage was 4.7% in Southern China and 3.9% in Northern China. Across 125 paired weeks, Southern China had a mean positivity 4.41 percentage points higher than Northern China (95% CI, 2.23–6.59; P < 0.001) and a mean ILI percentage 0.77 points higher (95% CI, 0.48–1.05; P < 0.001) (Table 3). The paired-week estimate is not the simple difference between the specimen-weighted overall positivities (14.6% and 13.3%), because each epidemiological week contributed equally to the paired analysis rather than being weighted by weekly specimen volume.

**Table 2:** Regional summary of influenza surveillance indicators.

Period	Region	Tested specimens	Positive specimens	Weighted positivity (%)	Mean ILI (%)	Peak positivity week	Peak positivity (%)
2024	Southern China	321,866	44,108	13.7	4.8	2024/W01	52.5
2024	Northern China	239,729	26,231	10.9	4.2	2024/W01	36.1
2025	Southern China	541,063	81,351	15.0	4.9	2025/W49	54.5
2025	Northern China	327,443	51,940	15.9	3.9	2025/W48	48.1
2026/W01-W21	Southern China	215,548	32,273	15.0	3.9	2026/W01	35.8
2026/W01-W21	Northern China	150,996	17,316	11.5	3.5	2026/W15	17.4
Overall	Southern China	1,078,477	157,732	14.6	4.7	2025/W49	54.5
Overall	Northern China	718,168	95,487	13.3	3.9	2025/W48	48.1

**Note.** Values for 2026 cover weeks 1–21 only. Peak weeks were identified from unrounded recalculated weekly positivity.

**Table 3:** Inferential and temporal comparison of weekly influenza activity between Southern and Northern China.

Indicator	Estimate	95% CI	P value	Method / interpretation
Weekly South – North positivity difference, percentage points	4.41	2.23 to 6.59	<0.001	Newey-West HAC, max lag 4
Weekly South – North ILI difference, percentage points	0.77	0.48 to 1.05	<0.001	Newey-West HAC, max lag 4
First-difference positivity cross-correlation	r = 0.781	--	--	Peak at lag + 1 week; positive lag indicates North leads South
First-difference ILI cross-correlation	r = 0.758	--	--	Peak at lag + 0 week; positive lag indicates North leads South

**Note.** Positive paired differences indicate higher values in Southern China. Cross-correlation was calculated on first-differenced weekly series; positive lag indicates Northern China leads Southern China.

Regional patterns varied over time. In 2024, weighted positivity was 13.7% in Southern China and 10.9% in Northern China; in 2025 it was 15.0% and 15.9%, respectively; and in 2026/W01-W21 it was 15.0% and 11.5%. During the largest winter wave, Northern China reached its positivity peak in 2025/W48 (48.1%) and Southern China in 2025/W49 (54.5%). In the first-differenced positivity series, the maximum cross-correlation occurred when Northern China led Southern China by one week (r = 0.781); first-differenced ILI changes were maximally correlated at lag 0 (r = 0.758), indicating strong synchrony in week-to-week syndromic changes (Table 3). Sensitivity analyses using Newey-West maximum lags of 8 and 12 weeks produced materially similar confidence intervals and retained statistical significance for both regional differences (all P < 0.001). The peak cross-correlation lags were also unchanged when the search window was restricted to +/-4 weeks or expanded to +/-12 weeks.

Testing volume varied substantially across weeks and was particularly low in Northern China during parts of the summer: the minimum northern weekly testing volume was 1,492 specimens in 2024/W31 and 2,272 in 2025/W18 (Fig 2). The public reports did not provide sufficient information to determine whether these denominator changes reflected sentinel-site operations, testing practice, reporting completeness or other surveillance processes. We therefore interpret raw counts together with positivity rather than as direct measures of population incidence.

**Fig 2:**
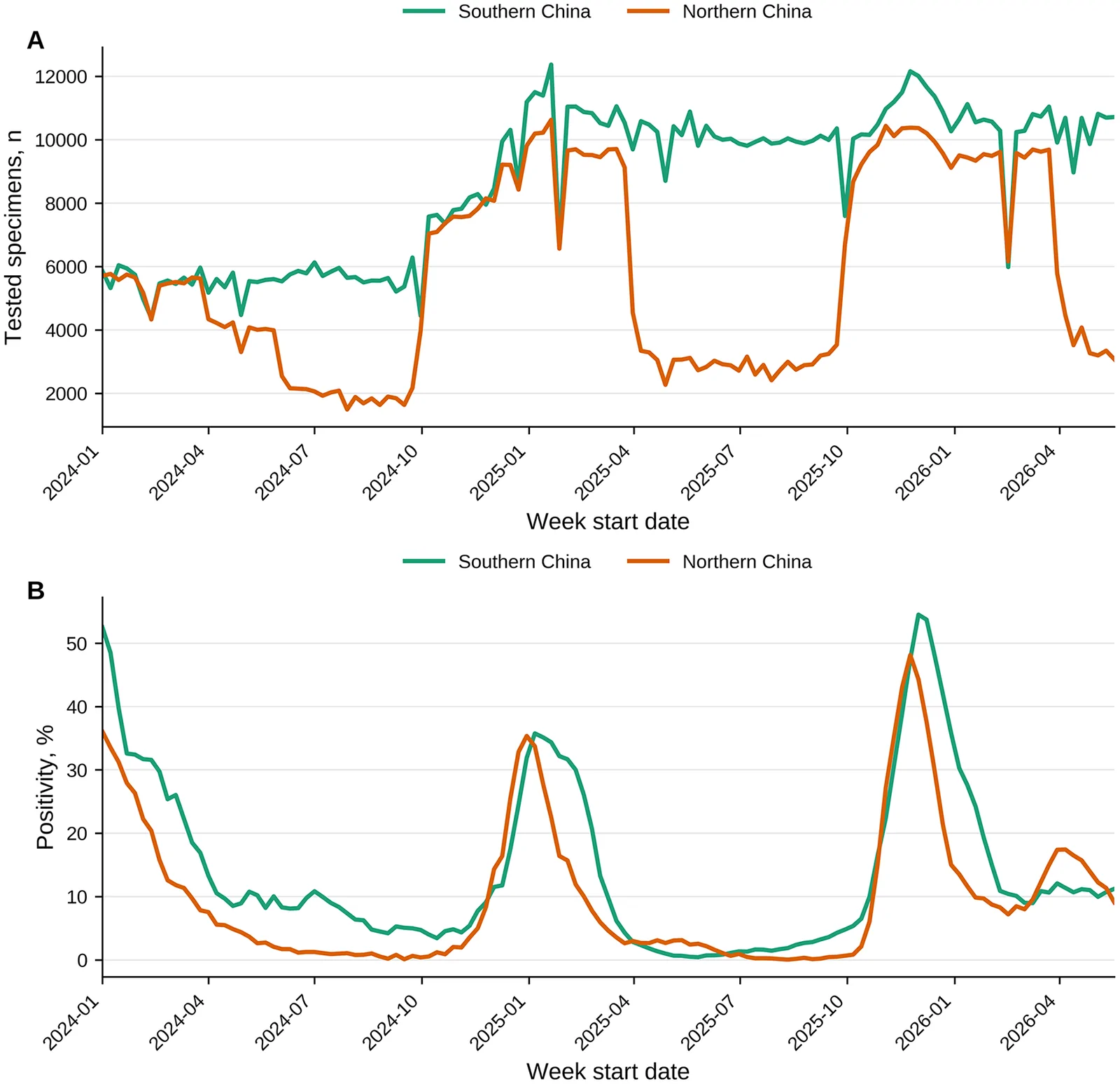
Regional heterogeneity of influenza activity. Panel A shows weekly tested ILI specimens in Southern and Northern China. Panel B shows weekly influenza positivity recalculated from reported positive and tested specimens in each region.

### National subtype and lineage replacement

National subtype/lineage composition changed substantially over time (Fig 3). After source-level reconciliation, 253,104 explicitly reported subtype/lineage detections were available. A(H3N2) was the largest overall detection category (115,336; 45.6%), followed by A(H1N1)pdm09 (85,518; 33.8%) and B(Victoria) (52,216; 20.6%). Five A-untyped and 29 B-untyped detections were explicitly reported; B(Yamagata) was not reported (Table 4). Because the official weekly reports occasionally contained incomplete or internally inconsistent subtype classification, the detection denominator was not forced to equal the 253,219 total influenza-positive specimens.

**Table 4:** National distribution of explicitly reported subtype/lineage detections.

Period	Reported detections	A(H1N1)pdm09	A(H3N2)	B(Victoria)	A untyped	B untyped	B(Yamagata)	Predominant subtype/lineage
2024	70,232	31,655 (45.1)	11,624 (16.6)	26,921 (38.3)	5 (0.0)	27 (0.0)	0 (0.0)	A(H1N1)pdm09
2025	133,283	53,692 (40.3)	77,748 (58.3)	1,841 (1.4)	0 (0.0)	2 (0.0)	0 (0.0)	A(H3N2)
2026/W01-W21	49,589	171 (0.3)	25,964 (52.4)	23,454 (47.3)	0 (0.0)	0 (0.0)	0 (0.0)	A(H3N2)
Overall	253,104	85,518 (33.8)	115,336 (45.6)	52,216 (20.6)	5 (0.0)	29 (0.0)	0 (0.0)	A(H3N2)

**Note.** Values are n (%). Percentages use the sum of explicitly reported subtype/lineage detections as denominator. The denominator may differ slightly from all influenza-positive specimens because official weekly reports occasionally contain incomplete or internally inconsistent subtype classification; no residual subtype counts were imputed.

**Fig 3:**
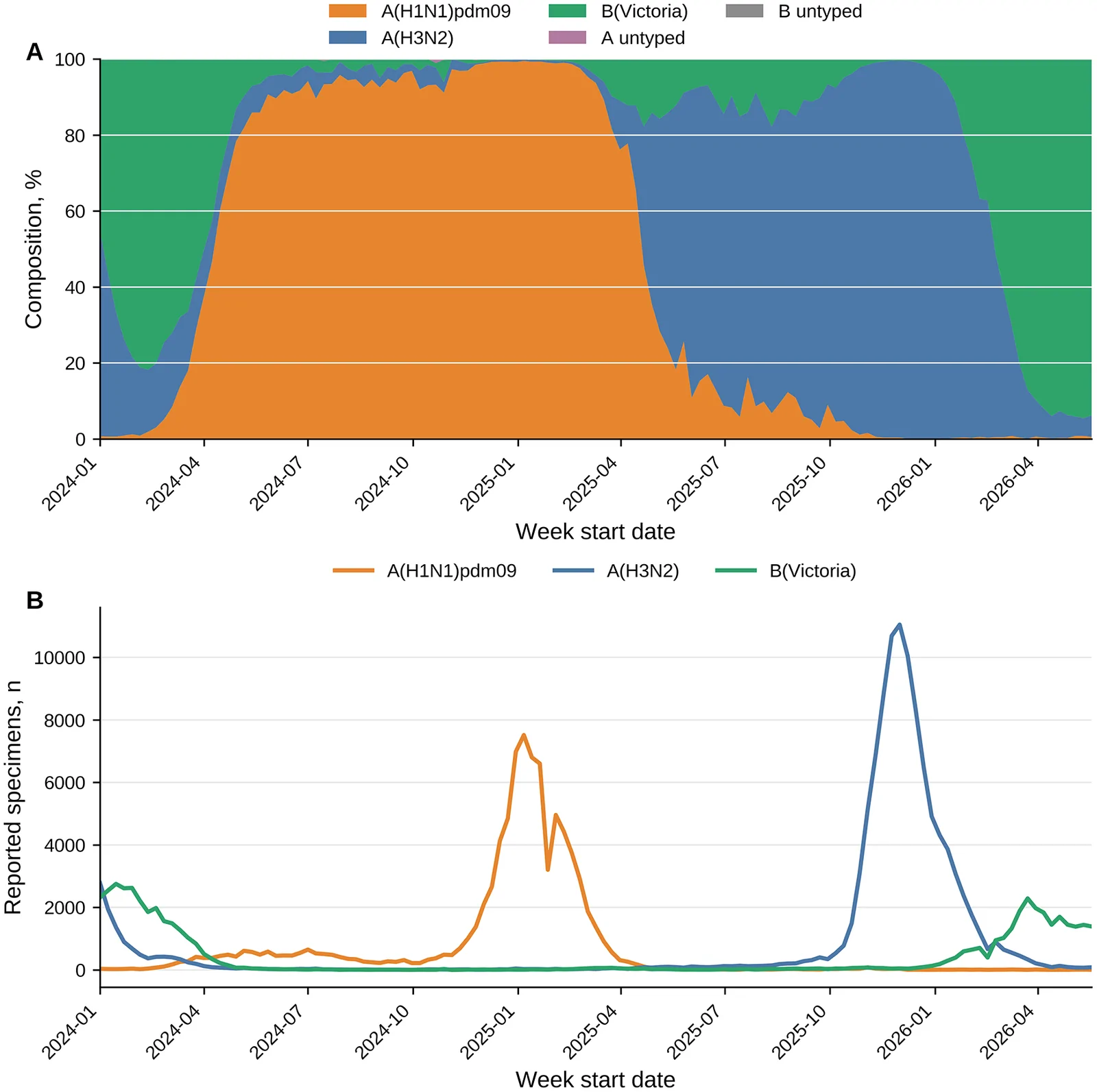
National subtype/lineage replacement. Panel A shows weekly proportional composition of explicitly reported national subtype/lineage detections, using the sum of reported detections as the denominator. Panel B shows weekly national counts of A(H1N1)pdm09, A(H3N2) and B(Victoria).

In 2024, A(H1N1)pdm09 was the largest annual detection category (45.1%), followed by B(Victoria) (38.3%) and A(H3N2) (16.6%). In 2025, A(H3N2) accounted for 58.3% of reported detections and A(H1N1)pdm09 for 40.3%. In 2026/W01-W21, A(H3N2) and B(Victoria) accounted for 52.4% and 47.3%, respectively (Table 4). To avoid visually overstating subtype proportions during weeks with very small regional denominators, regional subtype dynamics are presented as absolute reported counts rather than 100% composition (Fig 4).

**Fig 4:**
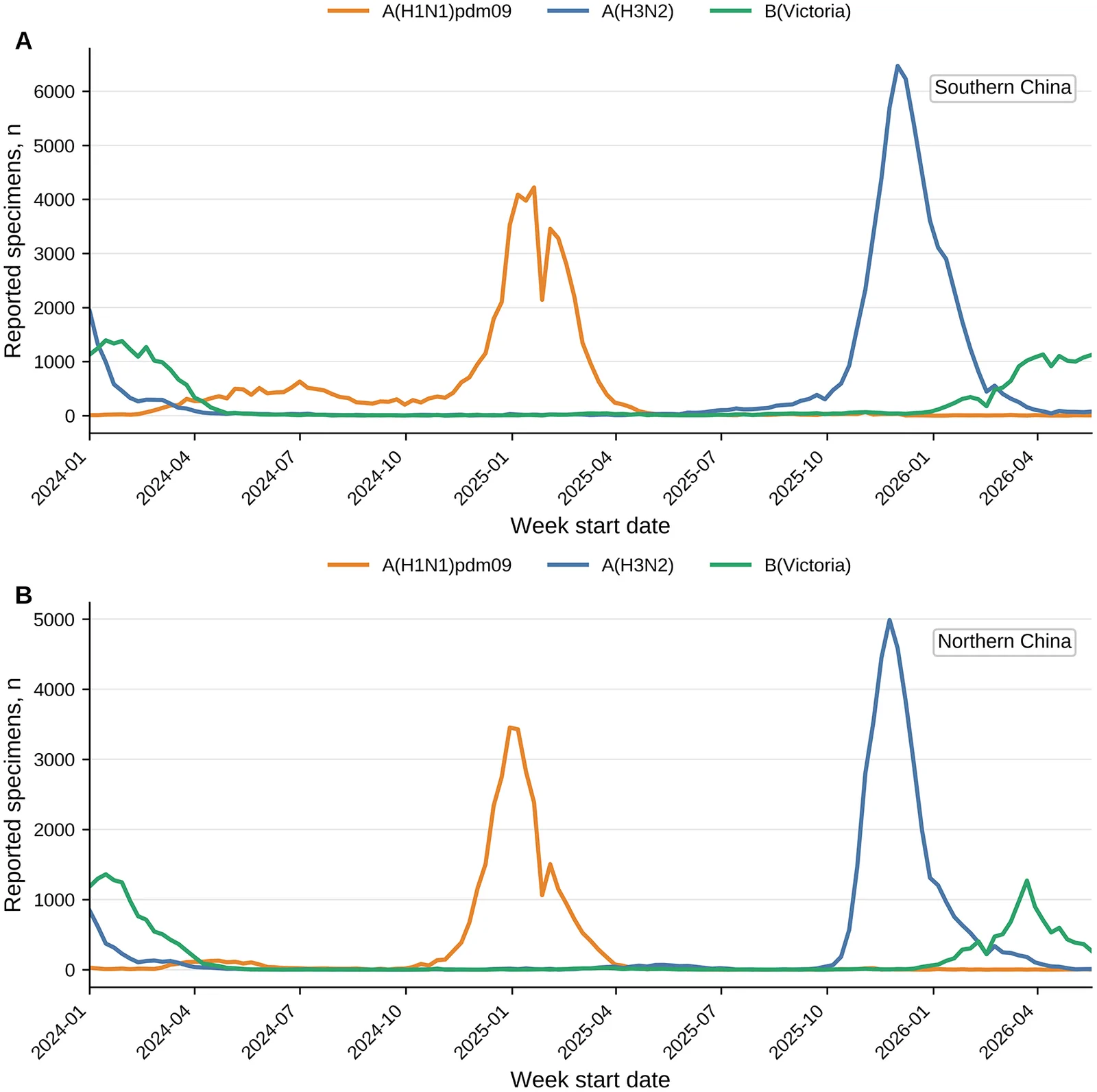
Regional counts of major influenza subtype/lineage detections. Panel A shows weekly reported counts of A(H1N1)pdm09, A(H3N2) and B(Victoria) in Southern China. Panel B shows the corresponding weekly reported counts in Northern China.

### Predominant subtype/lineage periods

Five national predominant subtype/lineage periods were identified (Table 5; Fig 5). A(H3N2) predominated in 2024/W01 only. B(Victoria) predominated from 2024/W02 to 2024/W14 (13 weeks), followed by A(H1N1)pdm09 from 2024/W15 to 2025/W17 (55 weeks). A(H3N2) then predominated from 2025/W18 to 2026/W08 (43 weeks), encompassing the largest winter wave. B(Victoria) predominated again from 2026/W09 through 2026/W21 (13 weeks).

**Table 5:** National predominant subtype/lineage periods.

Period no.	Start week	End week	Duration (weeks)	Dominant subtype	Cumulative influenza-positive specimens	Median positivity (%)	Peak week	Peak positivity (%)	Reported outbreaks, total
1	2024/W01	2024/W01	1	A(H3N2)	5,137	44.4	2024/W01	44.4	50
2	2024/W02	2024/W14	13	B(Victoria)	33,104	22.8	2024/W02	40.7	185
3	2024/W15	2025/W17	55	A(H1N1)pdm09	86,095	6.5	2025/W02	34.8	1,068
4	2025/W18	2026/W08	43	A(H3N2)	104,877	2.9	2025/W49	49.8	7,206
5	2026/W09	2026/W21	13	B(Victoria)	24,006	11.5	2026/W14	14.0	292

**Note.** Predominance was defined as the explicitly reported subtype/lineage detection with the largest weekly count. Periods were consecutive weeks with the same national predominant subtype/lineage.

**Fig 5:**
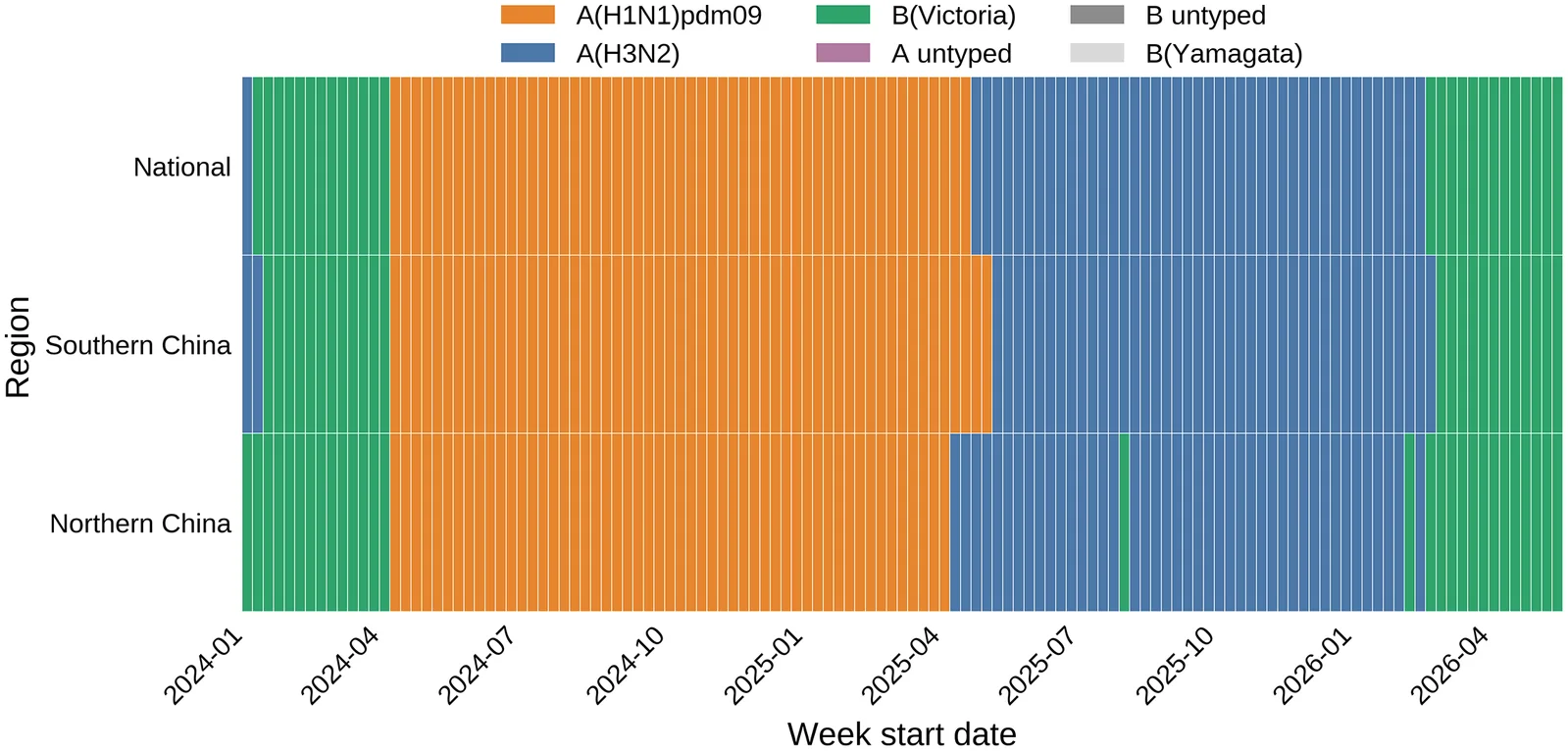
Dominant subtype/lineage heatmap. The heatmap shows the weekly predominant reported subtype/lineage in the national aggregate, Southern China and Northern China. Predominance was defined as the explicitly reported subtype/lineage detection with the largest weekly count in the corresponding analysis stratum.

The A(H3N2)-predominant period from 2025/W18 to 2026/W08 included 104,877 national influenza-positive specimens, the overall positivity peak in 2025/W49 and 7,206 reported outbreaks. This temporal co-occurrence does not establish causation but shows that the largest outbreak burden in the reconstructed series occurred during sustained A(H3N2) predominance.

### Influenza outbreak activity

A total of 8,801 influenza outbreaks were reported. Outbreak activity was concentrated in 2025, with 7,519 outbreaks compared with 903 in 2024 and 379 in 2026/W01-W21 (Table 6). Weekly outbreaks peaked in 2025/W48 at 1,541, one week before the national positivity peak (Fig 6).

**Table 6:** Reported influenza outbreaks by etiological classification.

Period	Total outbreaks	A(H1N1)pdm09	A(H3N2)	A untyped	B(Victoria)	B untyped	Mixed	Influenza negative	Pending
2024	903	506 (56.0)	38 (4.2)	13 (1.4)	81 (9.0)	29 (3.2)	60 (6.6)	106 (11.7)	70 (7.8)
2025	7,519	341 (4.5)	5,419 (72.1)	142 (1.9)	8 (0.1)	0 (0.0)	131 (1.7)	300 (4.0)	1,178 (15.7)
2026/W01-W21	379	11 (2.9)	60 (15.8)	1 (0.3)	210 (55.4)	7 (1.8)	5 (1.3)	21 (5.5)	64 (16.9)
Overall	8,801	858 (9.7)	5,517 (62.7)	156 (1.8)	299 (3.4)	36 (0.4)	196 (2.2)	427 (4.9)	1,312 (14.9)

**Note.** Values are n (%). Values for 2026 cover weeks 1–21 only.

**Fig 6:**
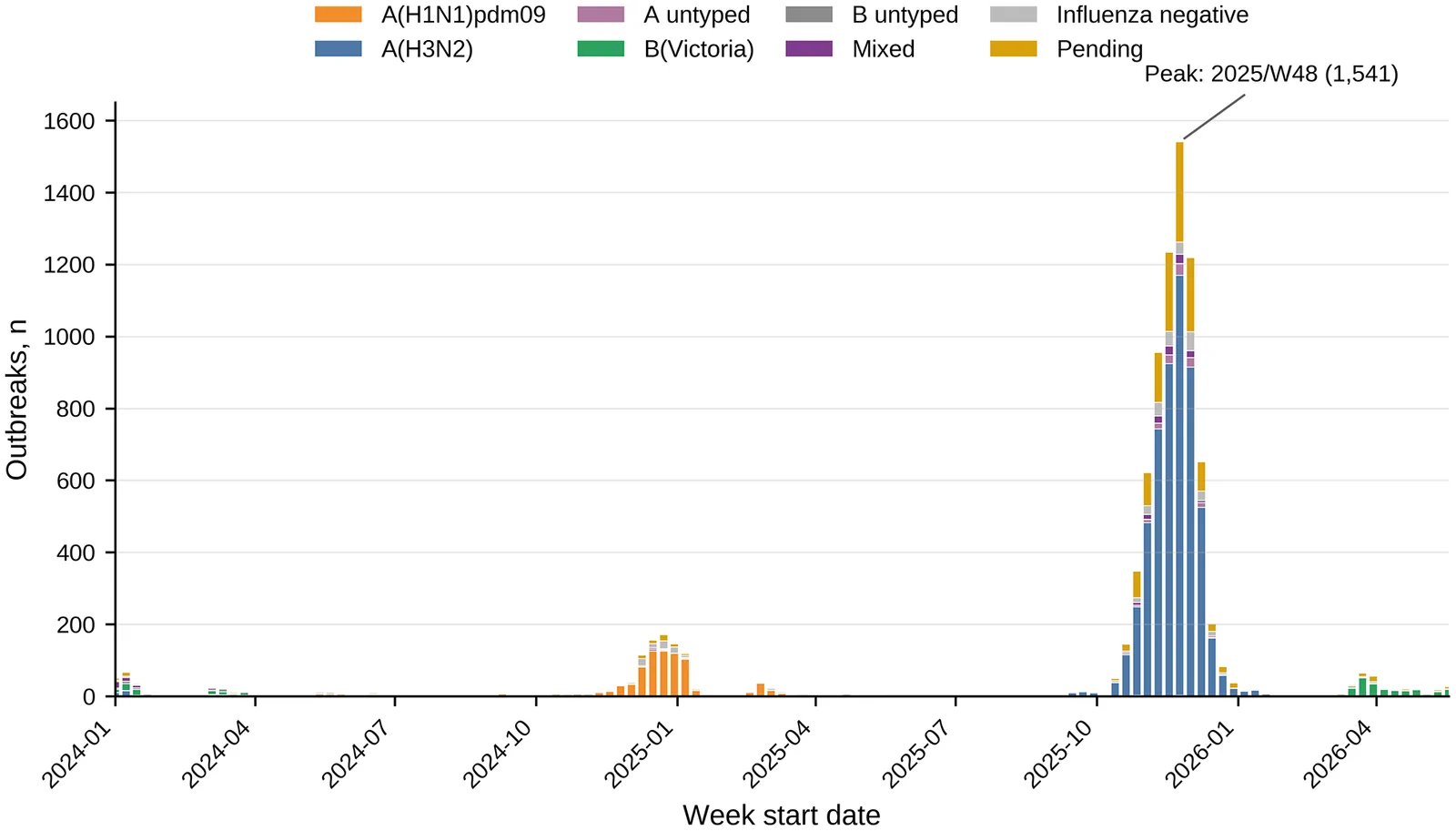
Weekly influenza outbreak activity by etiological classification. Stacked bars show raw weekly numbers of reported influenza outbreaks by etiological classification from 2024/W01 to 2026/W21. Categories include A(H1N1)pdm09, A(H3N2), influenza A untyped, B(Victoria), influenza B untyped, mixed influenza viruses, influenza-negative testing and pending etiology.

Overall, A(H3N2)-associated outbreaks were the largest etiological category (5,517; 62.7%), followed by pending etiology (1,312; 14.9%) and A(H1N1)pdm09 (858; 9.7%). In 2024, A(H1N1)pdm09 accounted for 506 of 903 outbreaks (56.0%); in 2025, A(H3N2) accounted for 5,419 of 7,519 (72.1%); and in 2026/W01-W21, B(Victoria) accounted for 210 of 379 (55.4%) (Table 6). Fig 6 displays raw weekly outbreak counts; the previous square-root-transformed stacked presentation was removed because separately transforming categories before stacking obscured the relationship between stack height and total outbreaks.

### Data-quality audit

Field-level comparison of the two working datasets identified direct file-to-file discrepancies, while automated arithmetic checks and targeted source verification identified additional source-level inconsistencies, including national-regional mismatches and subtype totals that did not reconcile with type totals. The final dataset uses explicitly reported China CDC/CNIC values and does not impute residual subtype counts. Examples include the 2025/W33 national tested total (13,029) differing from the sum of Southern and Northern totals (13,038) and the 2024/W41 national H1N1 count differing from the sum of regional H1N1 counts. These discrepancies are small relative to the full dataset and do not alter the major surveillance conclusions, but they are documented to preserve reproducibility (S2 Table).

## Discussion

This revised analysis extends a descriptive reconstruction of Chinese weekly influenza reports by adding paired-week statistical inference, exploratory temporal lag analysis, an influenza-season sensitivity summary and an explicit source-level data audit. Across 125 weeks, Southern China showed higher average weekly positivity and ILI activity, while week-to-week positivity changes were most strongly aligned when Northern China led Southern China by approximately one week. These results quantify regional temporal heterogeneity rather than relying only on visual comparison of national curves.

The national series remained multi-wave over the 2.4-year observation window. We deliberately avoid interpreting this short interval as evidence that influenza has “lost seasonality.” Long-term Chinese studies have established substantial latitude- and climate-dependent seasonality [11–13] and post-pandemic multi-country studies have documented altered timing and subtype circulation [14,15]. Our W40-W39 sensitivity summary further shows why calendar-year totals should not be equated with influenza seasons: the complete 2024/W40-2025/W39 interval was relatively low-positivity and A(H1N1)pdm09-predominant, whereas the subsequent incomplete 2025/W40-2026/W21 interval contained the large A(H3N2) wave. The present data therefore support region-sensitive and week-specific interpretation, not a claim of permanent loss of seasonal structure.

Contemporary surveillance elsewhere shows that the post-pandemic subtype pattern was not uniform across countries. During the 2024/25 season in Europe, A(H1N1)pdm09, A(H3N2) and B/Victoria co-circulated, with A(H1N1)pdm09 predominating in multicountry surveillance [19]; Canadian sentinel surveillance likewise described a delayed A(H1N1) pdm09-predominant season with lower A(H3N2) co-circulation [20]. In contrast, our Chinese series underwent sequential replacement and subsequently a prolonged A(H3N2)-predominant period associated with the largest outbreak surge. These differences reinforce that post-pandemic influenza dynamics should not be generalized from a single national surveillance system.

Regional differences were also evident in the surveillance denominator. Northern weekly testing volumes fell markedly during parts of the summers of 2024 and 2025. Because the public reports do not document the operational cause of these fluctuations, we cannot attribute them to incidence, sentinel-site participation, testing policy or reporting practices. This limitation reinforces the need to interpret specimen counts together with positivity and to avoid treating surveillance testing volume as a direct population denominator. The paired-week analysis partially addresses this problem by comparing contemporaneous regional surveillance indicators rather than pooling all specimens as independent observations.

Sequential subtype replacement was a prominent feature. After an initial A(H3N2)-predominant week, B(Victoria), A(H1N1)pdm09, A(H3N2) and B(Victoria) predominated sequentially. Subtype interference, population immunity and antigenic evolution can alter the competitive balance among influenza viruses [12,21]. B(Yamagata) was not reported in our 2024–2026 series. This observation is consistent with recent literature describing the probable disappearance of B/Yamagata and its implications for surveillance and vaccine composition [22–24]. Importantly, uncertainty remains around whether formal extinction can be declared, so our data support continued surveillance rather than a definitive extinction claim.

The outbreak analysis links subtype ecology with operational surveillance signals. A(H3N2)-associated outbreaks accounted for 62.7% of all reported outbreaks and 72.1% of 2025 outbreaks; the weekly outbreak peak in 2025/W48 occurred during sustained A(H3N2) predominance and immediately before the national positivity peak. These aggregate data cannot identify causal pathways or individual sources of infection, but the alignment demonstrates that subtype-specific outbreak reporting adds information beyond national positivity alone and may help identify periods in which intensified antigenic and genomic characterization is most informative.

The data-quality audit is a further practical contribution. Public surveillance reports are often treated as internally consistent by default, yet a small number of weeks contained discrepancies between national and regional totals, type totals and subtype detections, or alternative report renderings. Rather than silently correcting those values, the revised analysis uses explicit source-level rules and documents the discrepancies in S2 Table. This approach is especially important for manually reconstructed surveillance series, because automated arithmetic closure can inadvertently convert an unclassified residual into a falsely labeled subtype category.

This study has important limitations. The public aggregate reports do not contain age, sex, vaccination status, comorbidities, severity, hospitalization, mortality or individual outcomes, so this analysis cannot address person-level risk or clinical burden. Province- or district-level data were not available in the reconstructed series, precluding granular spatial modeling, rural-urban comparisons and direct assessment of regional cultural behaviors such as masking. Meteorological conditions, school calendars, mobility and viral sequence data were also unavailable and may explain regional or subtype transitions. Testing volumes varied over time and may reflect surveillance operations in addition to underlying influenza activity. The 2.4-year series is too short for a robust long-term seasonal forecasting model and the exploratory cross-correlation analyses do not establish direction of transmission or causality. Future work should link national weekly reports to province-level surveillance, vaccination uptake, climate, mobility, clinical severity and genomic data.

## Conclusions

Between 2024/W01 and 2026/W21, influenza surveillance in China showed multi-wave activity, measurable regional temporal heterogeneity, sequential subtype/lineage replacement and a concentrated A(H3N2)-associated outbreak surge in late 2025. Southern China had higher average weekly positivity and ILI activity, while week-to-week positivity changes in Northern China preceded those in Southern China by approximately one week in exploratory lag analysis. The revised source audit also shows that reuse of public surveillance reports requires explicit rules for handling small internal and cross-level inconsistencies. These findings support weekly, subtype-specific and region-sensitive interpretation of influenza surveillance signals.

## Supporting information

S1 Dataset Revised aggregate weekly influenza surveillance dataset, China, 2024/W01-2026/W21. This dataset contains aggregate epidemiological week identifiers, ILI percentages, testing counts, influenza-positive specimens, recalculated positivity, subtype/lineage detections and outbreak classifications. Exact calendar dates, source-document identifiers and free-text notes have been removed from the public supporting dataset.
(XLSX)

S1 Table Data audit of identified source-level discrepancies and reconciliation rules. This table documents field-level differences between working datasets, official-report arithmetic inconsistencies and the final analytic handling used in the revised dataset.
(DOCX)

S2 Table Sensitivity summary using the W40-W39 influenza-season convention. The first and final windows are partial because the study period begins at 2024/W01 and ends at 2026/W21.
(DOCX)
