# Supplementary material for: Weekly dynamics, subtype replacement and regional heterogeneity of influenza activity in China, 2024–2026: A national sentinel surveillance analysis
Source: PLoS One. 2026 Nov 4;21(11):e0361588. doi: 10.1371/journal.pone.0361588 (PMC13734590; doi:10.1371/journal.pone.0361588)
Supplement: Supplementary file 1 [file pone.0361588.s002.docx]

**S2 Table. Data audit of identified source-level discrepancies and reconciliation rules.**

| **Report week** | **Issue** | **Official/source observation** | **Final analytic handling** |
| --- | --- | --- | --- |
| 2024/W17 | Residual subtype value | Official Table 1 reports A(unsubtyped)=0 in South, North and national totals. | Set northern and national A untyped to 0; no residual imputation. |
| 2024/W30 | Official internal arithmetic inconsistency | South A=481, H1=468, H3=15, A(unsubtyped)=0; national H1=485 while regional H1 sums to 487. | Retain each explicitly reported value at the corresponding analysis level. |
| 2024/W38 | Official internal inconsistency / alternative report rendering | Archived source fields are not mutually arithmetically consistent; A(unsubtyped)=0 in the source used. | Retain explicit archived-source values; no forced arithmetic reconciliation. |
| 2024/W41 | National-regional classification mismatch | South H1=287; North H1=38; national H1=220; national A(unsubtyped)=0. | National analyses use national fields; regional analyses use regional fields. |
| 2024/W43 | National-regional classification mismatch | Regional H1 sums to 379; national H1=375; national A(unsubtyped)=4. | Retain official national and regional fields separately. |
| 2024/W44 | Explicit A(unsubtyped) omitted from working files | Official Table 1 reports South and national A(unsubtyped)=1. | Restore the explicit official value of 1. |
| 2025/W12 | Residual subtype value | Official Table 1 reports A(unsubtyped)=0 in South, North and national totals. | Set northern and national A untyped to 0; no residual imputation. |
| 2025/W28 | Missing H3 counts in earliest downloaded file | Official report: South H3=104, North H3=15, national H3=119. | Use official H3 values; predominant subtype=A(H3N2). |
| 2025/W33 | National-regional testing mismatch | South tested=10,043; North=2,995; national=13,029; regional sum=13,038. | Use national official value for national analyses and regional official values for regional analyses. |
| 2025/W52 | Week-start date typo | Official week spans 2025-12-22 to 2025-12-28. | Correct internal plotting date; public S1 uses epidemiological week only. |

**Note.** No weekly observation was excluded. Small official inconsistencies were retained transparently rather than silently reconciled.
