# Supplementary material for: Weekly dynamics, subtype replacement and regional heterogeneity of influenza activity in China, 2024–2026: A national sentinel surveillance analysis
Source: PLoS One. 2026 Nov 4;21(11):e0361588. doi: 10.1371/journal.pone.0361588 (PMC13734590; doi:10.1371/journal.pone.0361588)
Supplement: Supplementary file 2 [file pone.0361588.s003.docx]

**S3 Table. Sensitivity summary using the W40-W39 influenza-season convention.**

| **Influenza-season window** | **Completeness** | **Weeks** | **Tested specimens** | **Positive specimens** | **Weighted positivity (%)** | **Predominant reported subtype/lineage** | **Reported outbreaks** |
| --- | --- | --- | --- | --- | --- | --- | --- |
| 2024/W01-2024/W39 | Partial | 39 | 358,086 | 50,991 | 14.2 | B(Victoria) | 330 |
| 2024/W40-2025/W39 | Complete | 52 | 806,385 | 77,719 | 9.6 | A(H1N1)pdm09 | 1,033 |
| 2025/W40-2026/W21 | Partial | 34 | 632,165 | 124,509 | 19.7 | A(H3N2) | 7,438 |

**Note.** 2024/W01-2024/W39 and 2025/W40-2026/W21 are partial windows; 2024/W40-2025/W39 is complete.
